# Are There Similar or Divergent Transitions to Adulthood in a Mediterranean Context? A Cross-National Comparison of Italy and Spain

**DOI:** 10.5964/ejop.v12i1.1043

**Published:** 2016-02-29

**Authors:** Ugo Pace, Marco Cacioppo, Valentina Lo Cascio, Giovanni Guzzo, Alessia Passanisi

**Affiliations:** a Faculty of Human and Social Sciences, Kore University of Enna, Enna, Italy; b Department of Human Sciences, Lumsa University of Rome, Rome, Italy; c Department of Economics, Business and Finance, University of Palermo, Palermo, Italy; Academy of Special Education, Warsaw, Poland

**Keywords:** transition to adulthood, identity status and attachment styles, Mediterranean model, cross-cultural, multiple correspondence analysis

## Abstract

The purpose of this study was to examine the differences and similarities between Italy and Spain in regard to emerging adults’ perceptions of identity status, autonomy, attachment, and life satisfaction. The goal was to verify whether a Mediterranean model of transitions from adolescence to adulthood exists. Three hundred and forty undergraduate students (171 Italians and 169 Spanish) ranging in age from 19 to 22 completed measures of identity status, emotional autonomy, attachment style, and life satisfaction. Multiple correspondence analyses provided a graphic synthesis of results. The results indicate that no common model of young adult development exists in Spain and Italy and that Italian youth have a more complex quality of development compared to their Spanish peers.

## Introduction

In times of large sociohistorical changes in Europe (see Adamski, 1994, for a review), several empirical studies have examined the transitions to adulthood across countries and cultures (e.g., Bang & Montgomery, 2013). Young adults are in the process of exploring a variety of possible life directions, relating to new relationships, different identity models, and autonomy from parental bonds (Arnett, 2014). Starting with David Reher’s (1998) study, researchers began to advance the idea that specific patterns of family and transitions to adulthood exist. In particular, Scabini (2000) identified three different models: the Nordic model, the British model, and the Mediterranean model. The Northern European model is marked by a long interim period between moving out of one’s parental home and starting a family. Young men leave the parental home at 26 years, young women at 21 years, and, on average, the first marriage takes place six and eight years later (Buhl, 2007; Weick, 2002). The British model is characterized by a premature separation from family of origin and an early marriage; however, the choice of parenting in this model is postponed, the couple remaining childless for a long time. Finally, in the Mediterranean countries, the term family indicates a diffuse network of relationships, obligations, and loyalties. The boundaries between households and kin networks are often considered not only in emotional and affective terms but also in legal and practical ones (Naldini, 2005). Moreover, household size is much larger, and a high percentage of the population lives in expanded households. In fact, in their late teens or early 20s, many youngsters live with their parents, either full time or part time, and more and more young people continue to do so until late in their 20s or even their 30s (Beyers & Goossens, 2008; Laudani, Guzzo, Lo Cascio, Pace, & Cacioppo, 2014; Pirrone, Nicolosi, Passanisi, & Di Nuovo, 2015). Not only do late adolescents live with their parents very often, they also refer to their parents as important sources of support (Dotterer, Hoffman, Crouter, & McHale, 2008). Furthermore, the definitive departure of young people tends to coincide somewhat closely with their marriage and finding a stable job. The years between adolescent maturity and marriage are spent largely within the parental household; generally, marriage does not even enter the picture unless it is accompanied by a corresponding emancipation from the parental home and the formation of a new household (Laudani et al., 2014).

Despite the large number of speculative studies conducted in the social sciences, little empirical evidence is available in the psychological sciences to support the existence of the Mediterranean model of transitions to adulthood (Buhl & Lanz, 2007). For this reason, the main aim of this study is to empirically verify whether the Mediterranean model of transitions to adulthood really does exist. This will help researchers understand how young adults develop their individuality in a proximal and distal context characterized by postponement of parental home leaving, marriage, or cohabitation. It will also help researchers to understand the difficulties of young people, especially females, when it comes to finding a stable job, especially in the absence of vocational training and with the strong protection of workers who are already employed (Buchmann & Kriesi, 2011; Tsani, Paroussos, Fragiadakis, Charalambidis, & Capros, 2013). It could be important, in this sense, to compare psychological processes, such as autonomy, attachment styles, and identity building, and the satisfaction linked to these developmental variables, which are supposedly influenced by common cultural features and family traditions shared by Italy and Spain. We argue that the results of the present study may help verify whether a psychological and not only a social and economic Mediterranean model of development really exists in the most populous countries of Southern Europe.

### The Impact of Culture on Developmental Processes

Toward the end of the second decade of life, young people are encouraged by significant others and their teachers to create action goals for their future lives in order to prepare for adulthood (see Zaleski, 1994, for a review). Successful transition to adulthood may be understood as the solving of three developmental tasks: finishing education, gaining employment, and establishing a partnership (Cacioppo, Pace, & Zappulla, 2013; Havighurst, 1953). As Nurmi (1993) suggested, it is often assumed that young people derive their goals from their experienced context, based on cultural representations of how and when to solve those developmental tasks. Some authors have recently found support for four basic transition patterns to adulthood in contemporary Europe. These can be conveniently labeled, according to welfare-regime typology, as social democratic (Scandinavian countries), liberal (the United Kingdom), conservative (Germany or the Netherlands), and Southern European (Mediterranean countries; Buchmann & Kriesi, 2011). According to authors, the social and welfare features of these regions contribute to the individual and psychological development of the people who live in those regions. In the social-democratic context, a developmental contest fosters young people’s autonomy and readiness for experimentation, thereby encouraging them to leave home early, form nontraditional unions, and become parents. The liberal context promotes young people’s self-reliance and autonomy, encouraging people to leave home early and make a strong push for employment in a competitive market. In the conservative context, family orientation does not particularly foster young people’s search for autonomy, which is revealed by the comparatively older age at which people leave home. Despite the relative ease of finding a job in this context, this does not translate into immediate marriage and parenthood. Finally, in the Southern European social and welfare context, strong hierarchically structured family ties, supported by the familial welfare regime, and the cultural legacy of a young man’s economic independence before embarking on marriage and parenthood, tend to greatly delay parental home leaving, union formation, and parenthood (Buchmann & Kriesi, 2011).

From the very beginning, the literature (Ryan & Lynch, 1989) has underlined that the construction of a sense of a personal self and the establishment of a healthy sense of independence, as revealed in tasks relating to identity and autonomy, are particularly salient in the adolescent period (Pace & Zappulla, 2009). However, in recent years, a small but growing literature has observed the development of these constructs from a cross-cultural perspective (e.g., Benson & Furstenberg, 2007). In 1963, Erikson stated that the formation of identity depends on the organization of prior experiences, which begin during adolescence, in a way that allows the individual to cope with new and challenging tasks. On the basis of this theory, social identities are constructed through interaction with significant others and are largely influenced by the social structures or contexts in which people exist (Benson & Kirkpatrick Johnson, 2009; Erikson, 1968). In this sense, several studies have argued that initial identity content is based on feedback youth receive from their parents, although youth will incorporate new information and contexts into their identities as they grow older (Pace & Zappulla, 2009, 2011). Thus, children’s experiences of their environment influence their subsequent behavior and characteristics. From a cross-cultural perspective, Goodnow (1988) stated that parents’ belief systems about child development are culturally rather than individually constructed. However, few studies have examined cross-national differences in identity formation during adolescence, and most of these studies have focused on comparing the United States and other nations (Crocetti, Schwartz, Fermani, Klimstra, & Meeus, 2012). Thus, further studies are needed to gain a better understanding of cross-national differences in identity formation in a Mediterranean context.

As previously mentioned, another important task of development is achieving emotional autonomy from one’s parents. Sometimes, this is considered to be one of the more controversial psychological constructs among researchers who describe the process of transformation of parent-child relationships during adolescent development (Pace & Zappulla, 2013). Based on controversial results, Beyers and colleagues (2005) proposed distinguishing between two types of emotional autonomy in relation to parents: a healthy separation from parents and a more conflictual and radical detachment from parents. Some scholars have seen autonomy as a culturally specific value, pertinent only to Western cultures (Markus & Kitayama, 1991). For instance, Rowland (1987) argued that in non-Western cultural contexts, where individual autonomy is not valued or promoted, the development of an autonomous identity is not a developmental goal. Based on this theory, many studies have investigated different cultural groups. Helwig (2006), for instance, showed that the developmental pathway toward autonomy is consistent across diverse cultures. Moreover, Goodnow and Collins (1990) showed that cultural norms and values influence beliefs about the appropriate timing of autonomy. Specifically, several studies have examined the effects of cultural beliefs by comparing the autonomy expectations of adolescents and parents from different cultures or ethnicities (Daddis & Smetana, 2005). The results of these studies have indicated that European and American adolescents and parents generally expect adolescents to attain autonomy at earlier ages than families from other ethnicities or cultures do, including Asian American early adolescents and parents (Feldman & Quatman, 1988), middle adolescents (15- to 18-year-olds) from Hong Kong, and 12-, 14-, and 16-year-old American adolescents of Mexican, Chinese, and Filipino backgrounds (Fuligni, 1998). Therefore, these studies have shown that larger macro-level contexts, such as political and economic trends, influence the issue of autonomy (Bronfenbrenner & Morris, 1998). Nevertheless, the debate about the universality of the construction of autonomy is not yet over (Soenens, Park, Vansteenkiste, & Mouratidis, 2012).

The third aspect pertains to the construct of attachment style. As suggested by Roisman and colleagues (2001), parental and peer attachment affects individuals from “the cradle to the grave” (p. 159), emphasizing the continuity of early attachment patterns into adult life (Pace, Cacioppo, & Schimmenti, 2012; Pace, Madonia, Passanisi, Iacolino, & Di Maggio, 2015). Since Bowlby (1973, 1980) carried out his studies of attachment, it has been argued that the functions and dynamic processes of attachment, which develop in relation to primary caretakers, have a significant degree of stability over time and across relationships. For this reason, early attachment relationships are expected to influence the way people regulate their subsequent interpersonal behaviors and emotions (Elicker, Englund, & Sroufe, 1992; Leanza, Lo Porto, Passanisi, & Leanza, 2013; Schimmenti, Passanisi, Gervasi, et al., 2014; Schimmenti, Passanisi, Pace, et al., 2014). Although some research studies on attachment (e.g., Grossmann, Grossmann, & Keppler, 2005) have supported the notion, found in Bowlby (1969), that the core components of attachment theory are culturally universal, several studies show that attachment cannot be generalized across all cultures (Baiocco, Pallini, & Santamaria, 2014); however, the results in this area are incoherent. Comparing the cultural values of the United States and Japan, Rothbaum and colleagues (2000) found that the cultural value of a child’s autonomy influences the meaning of the sensitive and responsive care given by parents: in Japan, early interactions are more emotion-based, whereas, in the United States, they are more information-based. Other authors have argued that the tenants of attachment theory reflect the values and meanings of Western culture, such as secure attachment and the construction of social competence.

### Aims and Hypotheses

In this study, I compare the cross-cultural similarities and specificities of the developmental processes and life satisfaction of emerging adults in Italy and Spain. I aim to examine the differences and similarities between these countries with regard to emerging adults’ perceptions of identity status, emotional autonomy, attachment style, and life satisfaction. The goal is to verify whether the Mediterranean model of transitions to adulthood exists. Specifically, I hypothesized that there would not be a significant difference between Italy and Spain in relation to the scores for all the variables in the study or the relationship between them. Usually, when research is carried out on the Mediterranean model, it focuses on the individualization process and the related family attitude, i.e., whether personal or economic autonomy among adolescents is encouraged and promoted or not. Therefore, it seems important, beyond speculation on the two countries’ common traditions, to conduct an empirical study on those variables that are considered to be relevant to the psychological emancipation of adolescents from their families.

## Method

### Participants

The study examined 171 Italian students (75 boys and 96 girls) who attended two universities in Italy and 169 Spanish students (70 boys and 99 girls) who attended two universities in Spain. Italian and Spanish undergraduate students (*N* = 340) ranging in age from 19 to 22 were recruited. In particular, the average age of the Italian students was 20.25 (*SD* = 1.80 years), whereas among Spanish students, the average age was 19.66 (*SD* = 1.67 years). Both the Italian and Spanish samples consisted only of Caucasian students. Moreover, the samples were comparable in terms of years and types of schooling, sex, and age group composition. Approximately 92% of the Italian and Spanish participants lived in two-parent households, whereas the remainder of participants lived with only one parent (usually the mother).

### Procedure

Participants completed consecutively self-report measures on developmental processes and life satisfaction. They also provided information on their age, gender, and ethnicity prior to completing questionnaires. Data were collected in two separate waves (data from Italy were collected first, then Spanish data). A standard informed assent was obtained before answering the questionnaire and students were informed that they could stop participation at any time. Research procedures described in this article were performed in compliance with the American Psychological Association, the Italian Psychological Association ethical guidelines for research and the ethical guidelines of the Spanish Psychological Society. The present research is part of a wider and complex study in which many other variables, particularly regarding the differential characteristics of family contexts between Italy and Spain, were taken into account. The results of the related research have been published in 2014 (Laudani et al., 2014). These related studies have tried to shed light on whether the individual and contextual aspects of adolescent development in Italy and Spain may be considered as similar or not.

### Measures

#### Ego Identity Process Questionnaire

The Ego Identity Process Questionnaire (EIPQ; Balistreri, Busch-Rossnagel, & Geisinger, 1995) is the most popular measure in Marcia’s (1966) identity status paradigm. In particular, the Author described four qualitatively different identity statuses that arise from the presence or absence of two psychological adolescent processes: “Exploration” and “Commitment” (Marcia, 1980). Using these two dimensions, adolescents are classified into one of the four identity statuses: *Achievement* is characterized by a period of active exploration leading to a firm identity commitment; *Foreclosure* is characterized by strong commitments without having explored other possible alternatives; *Moratorium* refers to adolescents’ active exploration of different alternatives without strong current commitments; *Diffusion* refers to adolescents who do not actively explore different identity alternatives and who lack strong identity commitments (Crocetti, Rubini, Luyckx, & Meeus, 2008). Specifically, EIPQ consists of 32 items within four ideological domains (values, occupation, religion, and politics) and four interpersonal domains (sex role, family, friendship, and dating) (Pace & Zappulla, 2009). The questionnaire was translated into Italian and Spanish and then back-translated by a native speaker. In the current study, the internal consistency (Cronbach’s α) for the domain scores in Italian and Spanish version ranged from .70 to .76.

#### Emotional Autonomy

The Emotional Autonomy Scale (EAS; Steinberg & Silverberg, 1986) was administered to assess the emotional autonomy constructs of separation and detachment. The scale consists of 20 items concerning four components of emotional autonomy: perception of parents as people, parental deidealization, nondependency on parents, and individuation. For each item, adolescents are asked to respond on a 5-point Likert-type scale ranging from 1 (strongly disagree) to 5 (strongly agree). The questionnaire was translated into Italian and Spanish and then back-translated by a native speaker. According to Beyers and colleagues (2005), an alternative factorial structure was considered, in which two higher-order factors substituted the original four factors. The first higher-order factor was called Separation (obtained by summing items of the original scales Deidealization and Nondependency and two items of Individuation). The second one was called Detachment (obtained by summing items of the original scale Perception of Parents as People and two items of Individuation) (Pace & Zappulla, 2009).

#### Relationship Questionnaire

Attachment style was assessed through the Relationship Questionnaire (RQ; Bartholomew & Horowitz, 1991; Italian version, Agostoni, 2007) was used to measure attachment style. Participants were asked to choose which of the four paragraphs - each prototypical of an attachment style (secure, dismissive, preoccupied, and fearful) - best represented themselves. The prototypes, along with sample sentences, are (a) Secure: “I feel comfortable depending on others and having others depend on me”; (b) Dismissing: “I am comfortable without close emotional relationships”; (c) Preoccupied: “I want to be intimate with others, but I often find that others are reluctant to get as close as I would like”; and (d) Fearful: “I find it difficult to trust others completely, or to depend on them”. Participants are asked to indicate on a 7-point scale how well each paragraph describes them (1 = It does not describe me at all, 7 = It very much describes me) (Pace, Cacioppo, & Schimmenti, 2012). The test-retest correlations of attachment dimensions are very high (on average, .78 for women and .86 for men) (Scharfe & Bartholomew, 1994). Prototype rating reliabilities (alpha) in this study ranged from .72 to .80.

#### Multidimensional Students’ Life Satisfaction Scale – abbreviated version

Life satisfaction was assessed through the Multidimensional Students’ Life Satisfaction Scale – abbreviated version (MSLSS - AV; Huebner, Zullig, & Saha, 2012; Italian version, Zappulla, Pace, Lo Cascio, Guzzo, & Huebner, 2014; Spanish version in long version, Galindez & Casas, 2011), a self-report scale that includes 30 items, which students could answer on a 4-point scale, ranging from 1 (never) to 4 (almost always). The MSLSS assesses satisfaction across five distinct domains, including family, friends, living environment, school, and self. A score for each domain was obtained by summing the individual items and dividing by the total number of items within the domain. Similarly, a general satisfaction score was calculated by summing all item scores and dividing by number’s item. The coefficient alpha for the total score has been reported as .92 (Huebner, 1991). In addition, the internal consistency scores for the domain scores in Italian and Spanish version ranged from .71 to .94 in previous studies (e.g., Zappulla et al., 2014).

### Data Analysis

We conducted preliminary analyses, including descriptive statistics and gender differences in the key study variables (ANOVAs). Distributions of all variables were checked for normality via kurtosis and skewness statistics. Moreover, a Multiple Correspondence Analysis (MCA) was performed in order to compare the cross-cultural similarities and specificities in the two countries, Italy and Spain, in regard to adolescents’ and emerging adults’ developmental factors. In particular, developmental processes and their correlates were entered as active variables, while country was introduced as supplementary variables in order to not affect the MCA solution, but still be able to know how the categories of this variable are positioned on the correspondence map. MCA is a special technique of exploratory factor analysis that graphically displays multivariate categorical data (Benzécri, 1992). Seen as a generalization of principal component analysis for categorical variables, it allows researchers to analyze the pattern of relationships in a complex data matrix by replacing raw data with a simpler data matrix (Abdi & Valentin, 2007). Associations between variables are examined by calculating the chi-square distance between variable modalities and between individuals. These associations can then be represented graphically by plotting the projection of the rows and the columns onto the dimensions extracted by the factor analysis. If two categories have similar count patterns, their profiles will be closer together in the correspondence map and they will have closer co-ordinates on dimensions that account for most of the variance. In summary, we selected MCA because it stems from a statistical strategy that makes it possible to simultaneously manage multiple variables within a given system. It has the advantage of plotting the similarities and differences across the countries on a comprehensive graphic. To conduct the MCA, continuous data for each of the variables of the study were reorganized into three categories: these categories represent values within 1 (low) and 3 (high). For instance, “family satisfaction 3” includes ratings above the third quartile on family satisfaction subscale, whereas “family satisfaction 1” includes ratings in the first quartile. Results will be limited to the interpretation of the first two factors extracted by the MCA (Ben Ammou & Saporta, 2003). Moreover, additional analyses were done to control the potential effect of disparities between the subsamples. We computed the MCA on SPSS software (Laudani et al., 2014).

## Results

Descriptive analyses for all independent and dependent variables are presented in Table 1. In order to analyze possible gender differences in the key study variables, we performed oneway analyses of variance (ANOVAs). They revealed no statistically significant country effects in the scores of all variables in the study.

**Table 1:** Descriptive Statistics for the Total Sample, for Spain and Italy

	Total			Spain^a^		Italy^b^	
Variables	*M*	*SD*	*SE*	*M*	*SD*	*M*	*SD*
Separation	3.17	0.58	0.13	3.19	0.56	3.14	0.60
Detachment	3.29	0.48	0.12	3.28	0.43	3.30	0.51
Exploration	1.96	0.25	0.11	1.96	0.25	1.96	0.24
Commitment	2.21	0.26	0.13	2.26	0.25	2.14	0.25
General satisfaction	3.96	0.46	0.14	4.08	0.42	3.85	0.46
Family satisfaction	3.90	0.76	0.12	4.01	0.72	3.80	0.79
Friends satisfaction	4.30	0.66	0.13	4.48	0.53	4.12	0.73
School satisfaction	3.94	0.59	0.11	3.96	0.55	3.93	0.62
Living environment satisfaction	3.75	0.69	0.13	3.98	0.60	3.53	0.70
Self-satisfaction	3.93	0.55	0.12	3.99	0.53	3.87	0.57

*Note. N =* 340.

^a^*n* = 169. ^b^*n* = 171.

**Figure 1:**
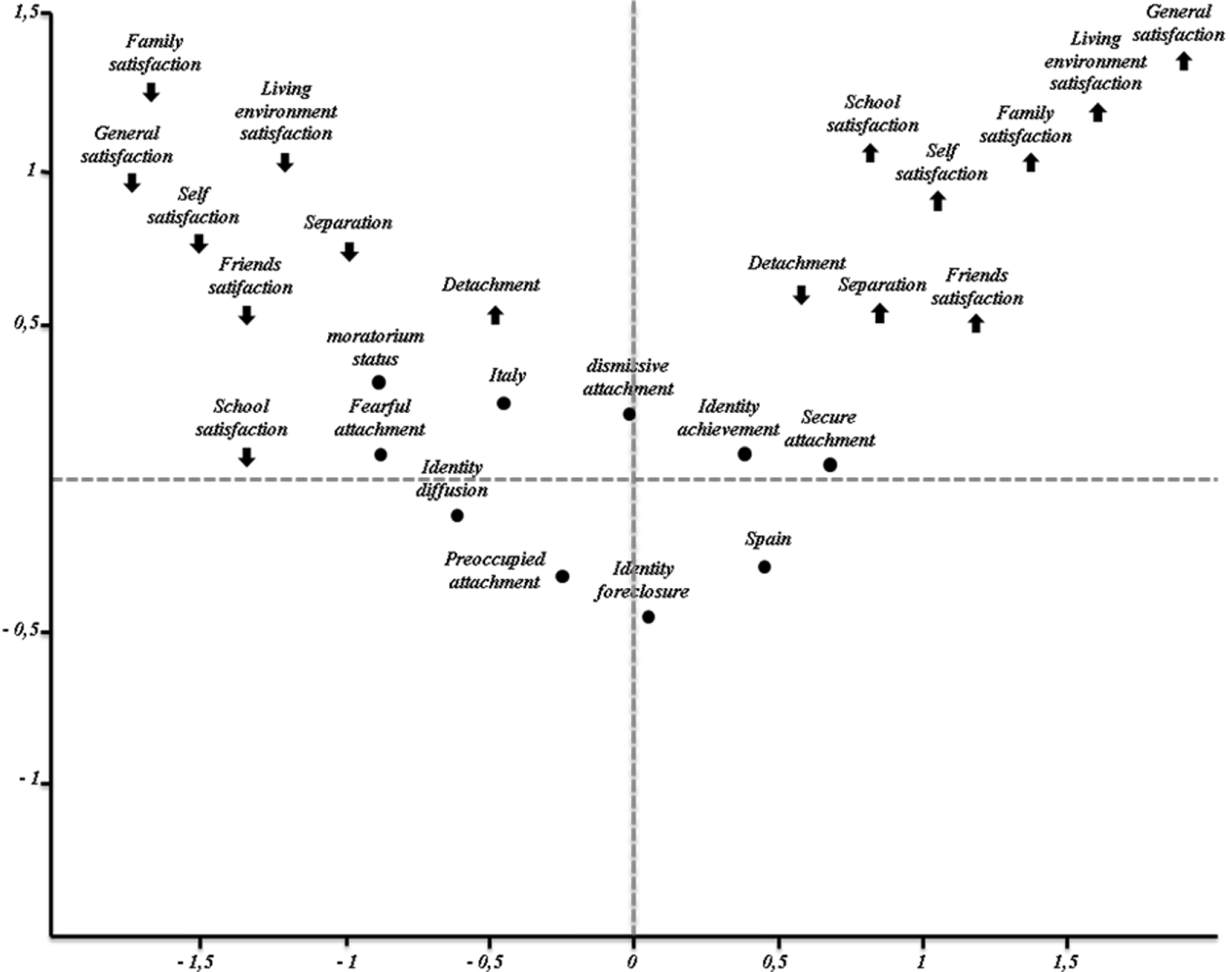
MCA two-dimensional space.

Figure 1 illustrates the MCA two-dimensional space that represents developmental processes and their correlates as active variables, while country as supplementary variables. MCA results are presented as follows: (1) interpretation of axes using contributions of the active variables to each factor; (2) interpretation of the position of the supplementary variable with respect to each factor; and (3) interpretation based on interpoint proximities in the two-dimensional map (Laudani et al., 2014).

The first axis (*λ*_1_) extracted by MCA, with an eigenvalue of *λ*_1_ = 3.32 and 32.23% of inertia explained, bipolarly encompasses the following measures of developmental processes at the negative pole (Table 2): low levels of domains of satisfaction (i.e., family, friends, living environment, self, and general life satisfaction), low separation, and high detachment. Moreover, it encompasses the following measures of attachment styles and identity status: preoccupied, fearful, and dismissing attachment styles, while moratorium, diffusion, and foreclosure identity status. At the opposite positive pole, the first axis encompasses the following measures of developmental processes: high levels of domains of satisfaction (i.e., family, friends, living environment, self, and general life satisfaction), high separation and low detachment. Moreover, it encompasses the following measures of attachment styles and identity status: secure attachment styles and achievement identity status. It is important to note that the analysis of discrimination measures has showed that the detachment dimension was not allocated to any of the obtained dimensions. Hence, this axis represents the interiorization of secure attachment models in developmental processes and satisfaction for different domains of life, as opposed to interiorization of insecure attachment models in developmental processes and low satisfaction for different domains of life.

**Table 2:** Summary of MCA Results

Negative Pole	Positive Pole
*First Axis* (λ_1_ = 3.32)	
Low level of Life Satisfaction (Family, Self, Friends, Environment) Low level Separation High level of Detachment Preoccupied Attachment Fearful Attachment Dismissing attachment Moratorium Diffusion Foreclosure High level of Life Satisfaction	(Family, Self, Friends, Environment) High level of Separation High level of Detachment Secure Attachment Achievement Identity
*Second Axis* (λ_2_ = 2.22)	
Lack of School Satisfaction	High Level of School Satisfaction

The second axis (*λ*_2_), with an eigenvalue of *λ*_2_ = 2.22 and 22.17% of inertia explained, bipolarly of high level of school satisfaction at the positive pole and low level of school satisfaction at the negative pole. Hence, this axis represents high satisfaction in school activities, as opposed to lack of satisfaction in school activities.

As for differences between countries, the two country-related profiles of categories are significantly different from the mean profile of the first axis [*t*(171) = 3.04; *p* < .05 for Italy and *t*(169) = -3.84; *p* < .05 for Spain]; moreover, they are significantly different from the mean profile for the second axis [*t*(171) = 2.75; *p* < .05 for Italy and *t*(169) = 2.17 *p* < .05 for Spain].

The profile for Italy falls in the top left quadrant of the graph, which is defined by the negative pole of the first axis and the negative pole of the second axis. This quadrant is characterized by profiles of low satisfaction for different domains of life, lack of individuation processes, and interiorization of insecure attachment models in developmental processes. Therefore, compared to Spain adolescents’ perception, Italian adolescents perceive low levels of all domains of satisfaction (i.e., family, friends, living environment, school, and self) and low separation. Moreover, they experience less advanced identity status - i.e., foreclosure, moratorium and diffusion – and they report preoccupied, fearful, and dismissing attachment styles.

Conversely, the Spain profile is characterized by categories high in satisfaction for different domains of life, good in individuation processes, and good in interiorization of secure attachment models. Thus, compared to Italian adolescents’ perception, Spain adolescents perceive high levels of all domains of satisfaction (i.e., family, friends, living environment, school, and self) and high separation. Moreover, they experience more advanced identity status - i.e., achievement – and they report secure attachment styles.

## Discussion and Conclusions

The purpose of this study was to examine the differences and similarities between Italy and Spain with regard to emerging adults’ perceptions of identity status, emotional autonomy, attachment style, and life satisfaction. The goal was to verify whether a Mediterranean model of transitions from adolescence to adulthood exists. In particular, we aimed to explore the existence and meaning of the so-called Mediterranean model of adolescent development through empirical evidence, as opposed to the speculative reasoning characteristic of the social sciences.

Regarding the primary analyses, the data we collected revealed no statistically significant effect of country in the scores of all the variables in the study.

Interesting findings emerged from the multiple correspondence analysis (MCA), which detected two axes of structured variables relating to the autonomy, attachment, identity, and well-being perceived by all adolescents in the two countries. The MCA results highlight the fact that young Italian adults, compared to their Spanish peers, perceive low levels of all domains of satisfaction (i.e., family, friends, living environment, school, and self) and low separation. Moreover, they experience less advanced identity status, i.e., foreclosure, moratorium, and diffusion, and report preoccupied, fearful, and dismissive attachment styles. The profile of the Spanish adolescent, conversely, is characterized by the following: a high level of satisfaction in different domains of life, a good level of satisfaction in terms of individuation processes, and a good level of satisfaction in terms of the interiorization of secure attachment models.

The data we collected were extremely surprising. Two groups of young adults, homogeneous for gender, age, and level of education (all the adults were in the first year of their university courses in social studies and humanities), described two completely opposite transitions from adolescence to young adulthood. What makes the findings completely reliable with the psychological literature is the absolute consistency of the results within the two groups. In fact, if we consider the relationship between psychological variables related to personal development and well-being, it is possible to make the following claim: when adaptive separation from parents characterizes the growth process, the quality of attachment and identity is positive, based on high levels of exploration and commitment within adolescent developmental tasks; meanwhile, personal satisfaction in all domains can be considered adequate. One result that contradicts the research hypothesis is the fact that the description of this consistent set of data describes only the Spanish young adults. The set of data that characterizes the Italian young adults is completely different but also consistent. In terms of the maladaptive characteristics of young people’s autonomy from their parents, which was assessed as ambivalent, identity appears to be inconsistent and is not focused on developmental tasks. Finally, among Italian youths, the models of attachment are more disadaptive than those of their Spanish peers and are linked to low levels of personal satisfaction.

In this sense, it seems that belonging to a different nationality can be considered the only variable that affects the developmental trajectories of the subjects who participated in the present study: in particular, the data highlighted how Spanish adolescents show developmental trajectories characterized by a mature model of emancipation from childhood (autonomy, attachment, and identity) and consequent high levels of well-being (satisfaction in all domains of life). In contrast, Italian young adults show ambivalent patterns of development and consequent low levels of well-being.

A cultural approach would claim that individuals classify reality in terms of salient acts or events, making use of prototypical representations drawn from the popular psychology of their country or culture (Smorti, Menesini, & Smith, 2003). The present findings, in this sense, are underlined by the following fact: a constellation of meanings linked to the culture of belonging influence the representation of the emotional aspects characterizing the stage of emancipation from patterns of parent-child relationships, where emotional dependency is the frame of reference.

The results of the research clearly show that the transition to adulthood is actually different between the two countries, especially if we consider three of the major developmental tasks that characterize this stage of life: the development of emotional autonomy from parents, the transformation of attachment relationships, and the structuring of an identity based on the exploration of talents and the commitment to the pursuit of goals.

The first thought that comes to mind is the fact that the differences between Spanish and Italian young adults with regard to their level of well-being are consistent with the different ways of dealing with developmental tasks described above. It seems that, at the same age and level of culture, Spanish young adults have achieved a level of emotional maturity that exceeds that of their Italian peers. This maturity seems to affect both their perception of their relationship with their parents and, as a result, their perspectives on life and the actions they are willing to perform. The explanation for such a marked difference is extremely complex. What this puts into question, however, is the existence of a unique model of the Mediterranean’s transition from adolescence to adulthood.

The data of the present research—despite all the limitations associated with the methodology of the self-reported survey and the involvement of a single age group (late adolescents/young adults)—clearly indicate two things: no common model of adolescent development in Spain and Italy exists, and Italian youth have a more complex and ambivalent quality of development compared to their Spanish peers. Although we conducted the present research only among adolescents belonging to two Mediterranean countries, it is important to mention that the population of Italy and Spain accounts for almost 75% of the population of Mediterranean Europe (which traditionally includes Greece, Albania, the countries that make up the former Yugoslavia, Cyprus, Malta, and southern France). Despite this, the conclusions of this study affirm that it is difficult to support the existence of a unique Mediterranean model of transition to adulthood. Conversely, it seems necessary to identify the social and cultural characteristics that differentiate the process leading the individuals of these two countries to build their individuality in such different ways. The fundamental question may be this: does Spanish culture encourage autonomous development of young people more than Italian culture? Based on the data of this research and information that can be found in the blog of any Erasmus student or young traveler throughout Europe, the answer can only be positive. A conclusive answer to this question would require a multidisciplinary analysis of welfare policies and the widening of the study sample to adolescents from all areas of Mediterranean Europe. The value attributed to the emancipation of youth and the willingness of families to promote the autonomy of their late adolescents would also have to be investigated in order to explain, beyond speculative reasoning without scientific validity, why such a clear difference exists between adolescent development in the Mediterranean area.
